# Phytochemical Analysis and Biological Activities of Essential Oils from Leaves and Stems of *Dicranopteris pedata* (Houtt.) Nakaike

**DOI:** 10.3390/molecules31193518

**Published:** 2026-10-02

**Authors:** Shu Qiu, Qinglei Han, Cenyue Xiao, Pengxiang Lai

**Affiliations:** Sdu-Anu Joint Science College, Shandong University, Weihai 264209, China

**Keywords:** *Dicranopteris pedata*, essential oil, antibacterial, synergistic, antibiofilm, cytotoxic

## Abstract

*Dicranopteris pedata* (Houtt.) Nakaike (family Gleicheniaceae) is a widespread fern species commonly found in tropical and subtropical areas and has long been employed in folk remedies for its various therapeutic properties. This study aimed to evaluate the phytochemical profiles and the cytotoxic, antibacterial, and biofilm-formation inhibition activities of essential oils from *D. pedata* leaves and stems, as well as their synergistic interactions with conventional antibiotics. A total of 48 and 40 components were identified by GC-FID and GC-MS, respectively, accounting for 96.6% and 95.2% of the leaf essential oil (LEO) and stem essential oil (SEO). The main constituents were linalool, hexadecanoic acid, (*E*)-*β*-ionone, cyclohexadecanolide, (2*Z*,6*E*)-farnesol, *γ*-decalactone, *n*-nonanal, *cis*-linalool oxide (furanoid) and *α*-terpineol. The antibacterial activity was tested by microbroth dilution assays against four bacterial strains (Gram-positive: *Staphylococcus aureus* and *Bacillus subtilis*; Gram-negative: *Pseudomonas aeruginosa* and *Escherichia coli*). LEO showed a more potent antibacterial activity than SEO against the tested bacterial strains, with MIC values ranging from 20.00 to 640.00 μg/mL. In checkerboard assays, both LEO and SEO revealed strong synergistic activity with chloramphenicol or streptomycin against *Pseudomonas aeruginosa*. The EOs also exhibited inhibitory effects on the formation of *Staphylococcus aureus* biofilm, with IC_50_ values of 31.53 ± 4.53 μg/mL and 62.20 ± 9.36 μg/mL for LEO and SEO, respectively. Furthermore, the MTT assay revealed that LEO exhibited lower IC_50_ values (36.86 ± 1.97 to 67.34 ± 5.07 μg/mL) against HepG2, HCT-116, A-549, and MCF-7 cells, indicating greater cytotoxicity than SEO. Overall, both LEO and SEO exhibited antibacterial, antibiofilm and cytotoxic potential in these in vitro assays, with LEO generally outperforming SEO; however, further in vivo studies are needed before pharmaceutical or food-preservative use.

## 1. Introduction

Antimicrobial resistance represents a growing worldwide concern and has emerged as one of the greatest public health threats of the 21st century [1]. The WHO has identified AMR as a global priority, as the pipeline of new conventional antibiotics has stalled while resistant pathogens continue to spread [2]. The challenge is further compounded by the capacity of numerous pathogenic bacteria to develop biofilms, which are structured aggregates of microbial cells enveloped in an extracellular matrix of their own production [3,4]. Biofilms are notoriously difficult to eradicate, exhibiting up to 1000-fold higher tolerance to antibiotics compared with planktonic cells [5,6,7]. They are implicated in chronic infections, medical device contamination, and wound healing failure [8,9]. Consequently, there is an urgent need for novel antimicrobial agents with alternative mechanisms of action, including the ability to disrupt biofilms or act synergistically with existing drugs to restore their efficacy.

Plants have long served as a cornerstone of traditional medicine, with approximately 25% of modern pharmaceuticals derived from botanical sources [10,11]. Among these, secondary metabolites such as terpenoids, alkaloids, and phenolics exhibit diverse pharmacological properties, including antimicrobial [12], antioxidant [13,14], anti-inflammatory [15], antitumor [16,17], and insecticidal activities [18]. Essential oils (EOs), complicated combinations of volatile organic molecules derived from plants, have garnered significant attention due to their broad therapeutic potential and potentially fewer side effects compared with synthetic drugs [19,20,21,22]. For instance, the essential oil of *Melaleuca alternifolia* (tea tree oil) has demonstrated potent and broad-spectrum antimicrobial activity against various drug-resistant pathogens and has been widely formulated into commercial antiseptic and disinfectant products [23,24]. Meanwhile, the essential oil of Citrus limon (lemon) rind exhibits significant antiproliferative activity against cancer cells, with its major bioactive component, citral, shown to inhibit the proliferation of multiple cancer cell lines via cell cycle interruption and apoptosis induction [25].

*Dicranopteris pedata* (Houtt.) Nakaike, a fern of the family Gleicheniaceae, is extensively spread over tropical and subtropical areas of Asia and the Pacific [26]. This species has a longstanding history of application in traditional medicine, utilized for the treatment of numerous diseases such as hemorrhage, dysentery, and empyrosis, as well as microbial infections, inflammatory conditions, and fevers [27,28]. Previous phytochemical studies have characterized several classes of secondary metabolites in *D. pedata*, among which flavonoids, phenols, proanthocyanidins, and clerodane-type diterpene glycosides are prominent [28,29,30,31]. Despite its ethnopharmacological relevance, to the best of our knowledge, systematic investigations into the essential oil of *D. pedata* (DP-EO) have not been previously conducted. Moreover, no study to date has compared the chemical constitution and biological activities of the EOs derived from its leaves versus its stems, nor has any study evaluated their potential for biofilm inhibition or synergistic interactions with conventional antibiotics.

The present study was designed to address these questions through the following specific aims: (1) to characterize and compare the chemical composition of EOs hydro-distilled from the *D. pedata* leaves (LEO) and stems (SEO) utilizing GC-MS; (2) to assess their antibacterial potential against four pathogenic bacteria; (3) to assess their ability to inhibit biofilm formation; (4) to determine the synergistic effect of each EO when combined with conventional antibiotics using checkerboard assays and fractional inhibitory concentration (FIC) indices; and (5) to examine their cytotoxic activities on normal and cancer cell lines.

## 2. Results and Discussion

### 2.1. Chemical Constitution of the Leaves and Stems of D. pedate EO

**Figure 1:**
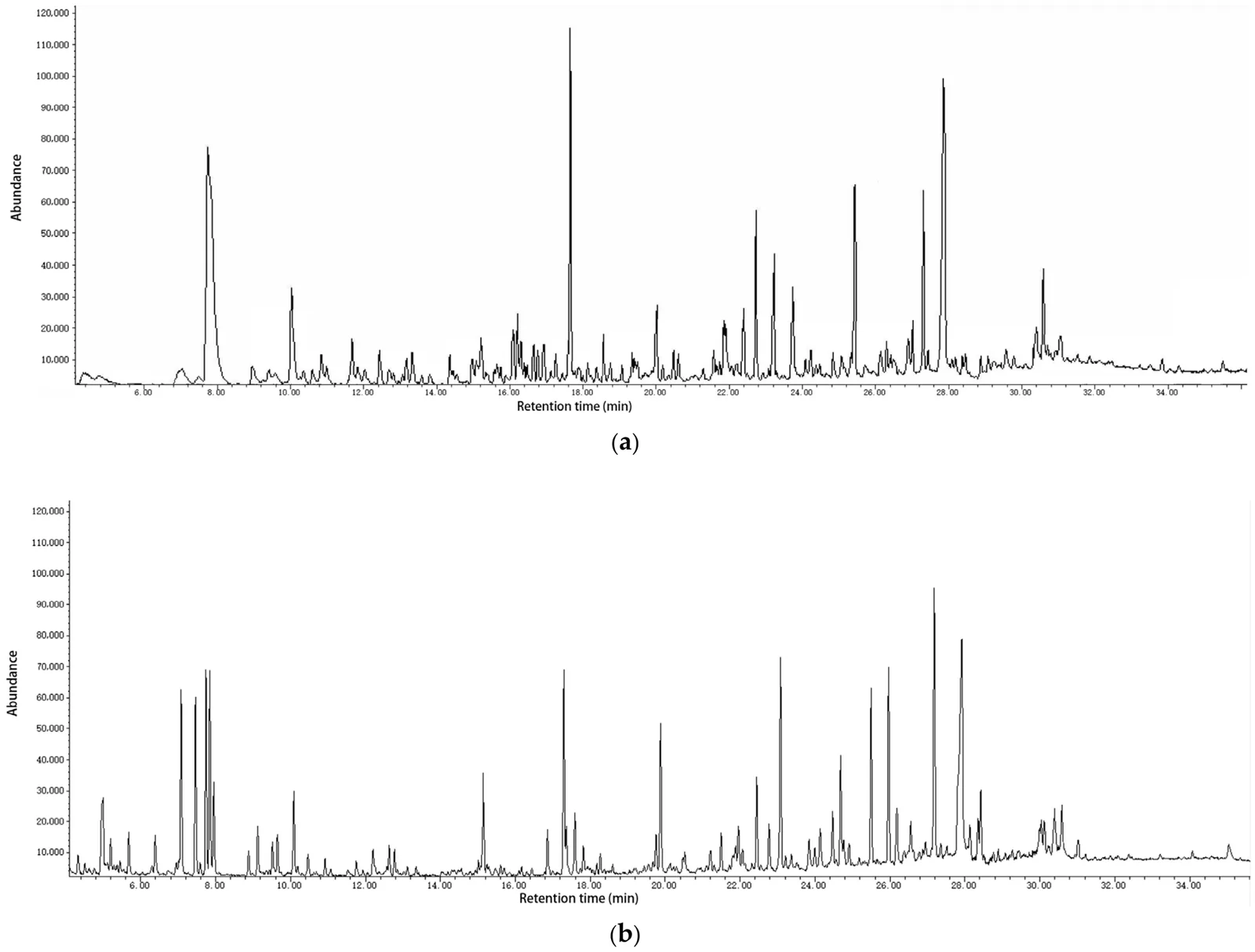
Chromatogram of GC–MS analysis of the essential oil of (**a**) *D. pedata* leaves; (**b**) *D. pedata* stems.

The EOs obtained from the leaves and stems of *D. pedate* yielded 0.11% and 0.07%, respectively. The composition of LEO and SEO was characterized by gas chromatography–mass spectrometry. All experiments were performed in triplicate. Forty-eight and forty chemical components were identified, and the identification rates of LEO and SEO were 96.6% and 95.2%, respectively (Table 1). The corresponding chromatogram is depicted in Figure 1a,b. The result shows that the main constituent classification of LEO was oxygenated monoterpenes (38.9%), followed by fatty acids and derivatives (22.6%) and oxygenated sesquiterpenes (14.6%). The main components of LEO were found to be linalool (21.0%), hexadecanoic acid (palmitic acid) (11.3%), (*E*)-*β*-ionone (7.0%), *α*-terpineol (4.0%), hexahydrofarnesyl acetone (3.9%), palmitoleic acid (3.8%), and *n*-pentadecanal (3.2%). In SEO, the main constituent classifications were fatty acids and derivatives (33.7%), followed by oxygenated monoterpenes (18.9%), carbonylic compounds (16.3%), and oxygenated sesquiterpenes (16.1%). The main chemical components identified in the SEO were hexadecanoic acid (palmitic acid) (12.3%), cyclohexadecanolide (6.3%), (*2Z,6E*)-farnesol (5.0%), linalool (4.5%), *γ*-decalactone (4.4%), *n*-nonanal (4.4%), *cis*-linalool oxide (furanoid) (4.1%), *trans*-linalool oxide (furanoid) (3.6%), caryophyllene oxide (3.6%), and hexahydrofarnesyl acetone (3.6%).

To our knowledge, the chemical constitution of EOs of the *Dicranopteris* genus has not been studied. Therefore, this work provides the first report on the volatile profile of *D. pedata* and the first comparative analysis between leaf-derived and stem-derived oils. The finding that leaves and stems exhibit distinct chemical profiles—with LEO being significantly richer in oxygenated monoterpenes (38.9% vs. 18.9%) and SEO showing higher contents of fatty acids and their derivatives (33.7% vs. 22.6%)—underscores the importance of organ-specific selection in essential oil research. Furthermore, it suggests that the conventional practice of using whole-plant material may overlook organ-dependent bioactivities.

Among the main components of DP-EO, linalool, a monoterpene that is present in various EOs, demonstrates a diverse range of biological activities, such as anxiolytic, antimicrobial, anti-inflammatory, and neuroprotective effects [32,33,34]. Its anti-inflammatory action is partly achieved by inhibiting the NF-κB signaling pathway, and it has shown neuroprotective potential in models of neurodegenerative diseases [35,36,37]. Palmitic acid, a prevalent saturated fatty acid also known as hexadecanoic acid, demonstrates a variety of bioactivities, such as anti-inflammatory, antioxidant, antimicrobial, and selective antitumor properties [38]. It exhibits antimicrobial activity against various pathogenic bacteria, including *Escherichia coli*, *Bacillus subtilis*, and *Staphylococcus aureus*, while its anti-inflammatory effect is attributed to the inhibition of key enzymes, specifically phospholipase A2 and cyclooxygenase (COX)-1/2 [39]. Furthermore, hexadecanoic acid exhibits selective cytotoxicity against cancer cells (e.g., leukemic cells) with minimal toxicity to normal cells, making it a promising lead compound for anticancer drug development [40]. In addition, several other major compounds in DP-EO, including (*E*)-*β*-ionone (7.0% in leaves), α-terpineol (4.0% in leaves), and caryophyllene oxide (3.6% in stems), are also worth noting. (*E*)-*β*-ionone has been reported to induce apoptosis in cancer cells and exhibit antimicrobial activity [41], whereas *α*-terpineol is known for its anti-biofilm and synergistic antibacterial properties [42], and caryophyllene oxide was found to exhibit anti-inflammatory, analgesic, and anticancer effects [43]. The presence of these compounds, together with minor compounds, may contribute to the overall antibacterial, synergistic, anti-biofilm and cytotoxic activities observed for DP-EO.

**Table 1:** Chemical components identified from essential oils of *D. pedata* leaves and stems.

Peak No.	Compound ^a^	RI ^b^	RI ^c^	Leaves (%)	Stems (%)
1	Hexanoic acid	971	967		3.1
2	Hexyl acetate	1011	1007		0.9
3	Benzene acetaldehyde	1042	1036		1.0
4	*cis*-linalool oxide (furanoid)	1070	1067	1.5	4.1
5	*trans*-linalool oxide (furanoid)	1087	1084		3.6
6	Linalool	1099	1095	21.0	4.5
7	*n*-Nonanal	1103	1100		4.4
8	Thujone	1107	1105		2.0
9	(3*Z*)-Nonen-1-ol	1153	1152	1.0	1.1
10	*cis*-linalool oxide (pyranoid)	1168	1170		0.8
11	*trans*-linalool oxide (pyranoid)	1173	1173		0.7
12	*α*-Terpineol	1191	1186	4.0	1.8
13	*β*-Cyclocitral	1222	1217	0.7	
14	Geraniol	1253	1249	1.5	
15	Nonanyl acetate	1309	1311	0.7	
16	(2*E*,4*E*)-Decadienal	1315	1315	0.9	
17	Dehydro-ar-ionene	1355	1355	0.3	
18	*α*-(*E*)-Ionol	1379	1376	0.6	
19	Hexyl hexanoate	1384	1382	0.7	2.2
20	*β*-Longipinene	1388	1398	1.3	
21	Dodecanal	1406	1408	0.8	
22	(*E*)-Caryophyllene	1424	1417	1.3	
23	(*E*)-*α*-Ionone	1429	1428	1.4	
24	*γ*-Maaliene	1433	1435	1.1	
25	*β*-Dihydroionol	1446	1449	0.8	
26	Geranyl acetone	1451	1451	0.6	
27	*α*-Humulene	1454	1455	1.0	1.0
28	*γ*-Decalactone	1472	1465		4.4
29	Germacrene D	1475	1480		1.2
30	(*E*)-*β*-Ionone	1485	1487	7.0	1.5
31	Tridecanal	1508	1509	0.6	
32	Cubebol	1526	1514	0.9	
33	Dihydroactinidiolide	1534	1535	0.4	
34	(*E*)-Nerolidol	1559	1561	0.6	
35	Dodecanoic acid	1563	1565	0.5	
36	Spathulenol	1577	1577		1.0
37	Caryophyllene oxide	1582	1582	1.8	3.6
38	Tetradecanal	1609	1611	0.7	
39	Junenol	1615	1618	0.7	
40	*α*-Bisabolol oxide B	1654	1656	0.8	0.9
41	Bulnesol	1671	1670	1.2	
42	*n*-Tetradecanol	1674	1671	1.7	1.3
43	2-Pentadecanone	1697	1697	1.5	2.1
44	*n*-Pentadecanal	1711	1711	3.2	1.3
45	(2*Z*,6*E*)-Farnesol	1727	1730	2.9	5.0
46	Tetradecanoic acid	1762	1758	2.5	1.0
47	(*Z*)-9-Hexadecenal	1775	1759	0.5	1.3
48	Ethyl tetradecanoate	1792	1795		1.4
49	Nootkatone	1803	1806		2.1
50	Hexadecanal	1812	1811	0.5	
51	Hexahydrofarnesyl acetone	1843	1847	3.9	3.6
52	*n*-Hexadecanol	1877	1878	0.6	1.5
53	(5*Z*,9*E*)-Farnesyl acetone	1885	1889	0.6	
54	2-Heptadecanone	1895	1902		1.7
55	(5*E*,9*E*)-Farnesyl acetone	1916	1913	1.2	
56	Methyl hexadecanoate	1921	1921	1.0	
57	Cyclohexadecanolide	1928	1933		6.3
58	Palmitoleic acid	1936	1933	3.8	
59	Isophytol	1944	1946	0.5	
60	Hexadecanoic acid	1966	1959	11.3	12.3
61	Hexyl dodecanoate	1977	1983		2.4
62	Manool oxide	1989	1987		1.2
63	Ethyl hexadecanoate	1992	1992		1.7
64	*n*-Octadecanol	2080	2077		1.6
65	Heptadecanoic acid	2084	2086		0.9
66	*γ*-Palmitolactone	2100	2104	1.1	1.5
67	Phytol	2110	2111	2.4	1.2
68	Oleic acid	2138	2141	1.0	
	Total identification			96.6	95.2

^a^ The compounds are ordered by their retention times on an HP-5MS column; ^b^ (retention index): RI-non-isothermal Kovats retention indices on an HP-5MS column relative to C_7_–C_30_ *n*-alkanes; ^c^ linear retention indices: by comparison of RI with those reported in the literature [44,45,46] on a non-polar column. Classes of compounds: oxygenated monoterpenes (No. 4–6, 8, 10–14, 23, 25, 26, 30, 33), sesquiterpene hydrocarbons: (No. 20, 22, 24, 27, 29), oxygenated sesquiterpenes (No. 32, 34, 36, 37, 39–41, 45, 49, 51, 53, 55), oxygenated diterpenes (No. 59, 62, 67), fatty acids and derivatives (No. 1, 2, 15, 19, 35, 46, 48, 56–58, 60, 61, 63, 65, 66, 68), carbonylic compounds (No. 3, 7, 16, 21, 28, 31, 38, 43, 44, 47, 50, 54), others (No. 9, 18, 42, 52,64).

### 2.2. Antibacterial Activity of the Leaves and Stems of D. pedate Essential Oils

The assessment of minimum inhibitory concentration (MIC) and MBC (minimum bactericidal concentration) values was conducted in three independent experiments utilizing the microdilution assay as described by [47,48]. The bacteria tested in the experiments comprised four strains: *Staphylococcus aureus* ATCC 6538, *Bacillus subtilis* ATCC 6633, *Pseudomonas aeruginosa* ATCC 27853, and *Escherichia coli* ATCC 25922. The MIC and MBC values of DP-EO are summarized in Table 2, which suggests that the EOs from *Dicranopteris pedata* leaves (LEO) and stems (SEO) exhibited antibacterial activity, with LEO generally more potent than SEO. Against *B. subtilis*, LEO showed strong activity (MIC = MBC = 20.00 μg/mL), indicating bactericidal action, while SEO was less active (MIC = 80.00 μg/mL, MBC = 160.00 μg/mL). For *S. aureus*, LEO (MIC = 160.00 μg/mL, MBC = 320.00 μg/mL) and SEO (MIC = 320.00 μg/mL, MBC = 640.00 μg/mL) were moderately effective, both with MBC/MIC ratios of 2, suggesting bactericidal effects. Unlike the two Gram-positive bacteria investigated, the EOs from *D. pedata* were less active against the tested Gram-negative bacterium. The LEO and SEO were bactericidal against *E. coli* (MIC = 640.00 μg/mL for both LEO and SEO), with MBC/MIC ratios of 1. For *P. aeruginosa*, the oils showed MIC values of 320.00 μg/mL (LEO) and 640.00 μg/mL (SEO), with MBC/MIC ratios of 4 (LEO) and 2 (SEO), indicating less bactericidal potential. However, chloramphenicol (the positive control) remained highly active against all strains (MICs ranging from 2.00 to 32.00 μg/mL). Overall, LEO outperformed SEO, and Gram-positive bacteria were more susceptible than Gram-negative ones.

The antibacterial activities of leaf essential oil (LEO) and stem essential oil (SEO) from *Dicranopteris pedata* correlate well with their chemical profiles. LEO, which exhibited lower MIC values against *B. subtilis* (20.00 μg/mL), *S. aureus* (160.00 μg/mL), and *P. aeruginosa* (320.00 μg/mL) compared with SEO, contained a notably higher proportion of oxygenated monoterpenes (38.9% vs. 18.9% in SEO). The major constituent linalool (21.0% in LEO vs. 4.5% in SEO) is well documented for its strong antibacterial and membrane-disrupting properties [49,50,51]. Additionally, *α*-terpineol (4.0% in LEO) and (*E*)-*β*-ionone (7.0% in LEO) have been reported to possess antimicrobial activity and contribute to the antibacterial activity of LEO [52,53,54]. In contrast, SEO’s lower content of these active oxygenated monoterpenes, together with a higher abundance of lactones (e.g., *γ*-decalactone, cyclohexadecanolide) and fatty acids, likely explains its weaker antibacterial effects [55]. The bactericidal effects of both oils (MBC/MIC ≤ 4) are likely attributable to the membrane permeabilization activity of their oxygenated monoterpene components, as previously characterized [56].

### 2.3. Results of the Checkerboard Method

The checkerboard method was performed to determine the combinatory efficacy of DP-EO and selected conventional antibiotics against four bacterial strains [57]. All experiments were performed in triplicate. The combined MIC and FICI (fractional inhibitory concentration index) values obtained from the DP-EO combination with chloramphenicol or streptomycin are summarized in Table 3 and Table 4, respectively. The findings were elucidated as follows: synergistic, FICI ≤ 0.5; additive, 0.5 < FICI ≤ 0.75; indifferent, 0.75 < FICI ≤ 2; no effect, FICI ≥ 2 [58]. The synergistic evaluation revealed that both leaf essential oil (LEO) and stem essential oil (SEO) from *Dicranopteris pedata* exhibited strong synergy with chloramphenicol (Chl) and streptomycin (Str), particularly against *P. aeruginosa* and *B. subtilis*. When combined with chloramphenicol, LEO possessed synergistic efficacy, with FICI values of 0.28, 0.25, 0.50, and 0.06 against *B. subtilis*, *S. aureus*, *E. coli*, and *P. aeruginosa*, respectively. The most pronounced effect was observed against *P. aeruginosa*, where the chloramphenicol MIC decreased from 32.00 µg/mL to 0.03 µg/mL, corresponding to a 1000-fold reduction under the conditions tested. The combination of SEO and chloramphenicol also displayed synergistic interactions against *B. subtilis* (FICI = 0.38), *S. aureus* (FICI = 0.31), and *P. aeruginosa* (FICI = 0.25), whereas the interaction against *E. coli* was at the synergy threshold (FICI = 0.50). In combination with streptomycin, LEO showed synergy against *B. subtilis* (FICI = 0.31), *S. aureus* (FICI = 0.28), and *P. aeruginosa* (FICI = 0.05), while the effect against *E. coli* was additive (FICI = 0.63). Similarly, SEO combined with streptomycin was synergistic against *B. subtilis* (FICI = 0.38), *S. aureus* (FICI = 0.38), and *P. aeruginosa* (FICI = 0.16), and additive against *E. coli* (FICI = 0.51). Overall, the combinations substantially reduced antibiotic MICs: in the presence of LEO, chloramphenicol MICs decreased 4- to 1024-fold and streptomycin MICs decreased 2- to 64-fold, whereas in the presence of SEO, chloramphenicol MICs decreased 4- to 16-fold and streptomycin MICs decreased 4- to 8-fold. The remarkable synergy observed could be attributable to LEO’s high content of oxygenated monoterpenes, especially linalool, which has been reported to disrupt bacterial cell membranes and increase permeability, facilitating antibiotic entry into bacterial cells [53]. In previous studies, linalool has been shown to synergize with ciprofloxacin against *Bacillus cereus* and *E. coli* and to exhibit bactericidal activity when combined with meropenem against carbapenemase-producing *Klebsiella pneumoniae* [59,60].

In the context of rising antimicrobial resistance and dwindling therapeutic options, synergistic combinations of essential oils (EOs) with conventional antibiotics offer a promising and pragmatic alternative to the development of entirely new drugs [61]. Such combinations can enhance the potency of existing antibiotics against resistant strains, making them effective where they were previously ineffective [62]. As this study has demonstrated with *Dicranopteris pedata* leaf essential oil (LEO), which reduced the minimum inhibitory concentration (MIC) of chloramphenicol against *P. aeruginosa* from 32 μg/mL to 0.03 μg/mL—a more than 1000-fold reduction—the potentiation effect can be noteworthy. This combination treatment requires less of the active pharmaceutical ingredient to achieve the same or better therapeutic effect, thereby reducing the toxic side effects caused by high-dose regimens. Furthermore, lower antibiotic doses alleviate the selective pressure on bacterial populations, thereby slowing the emergence of further resistance [63]. This study provides the first evidence of synergistic interactions between *Dicranopteris pedata* essential oils and conventional antibiotics, highlighting their potential as antibiotic adjuvants, especially against resistant *P. aeruginosa*.

### 2.4. Evaluation of Biofilm Formation Inhibition Activity Against Staphylococcus aureus ATCC 25923

**Figure 2:**
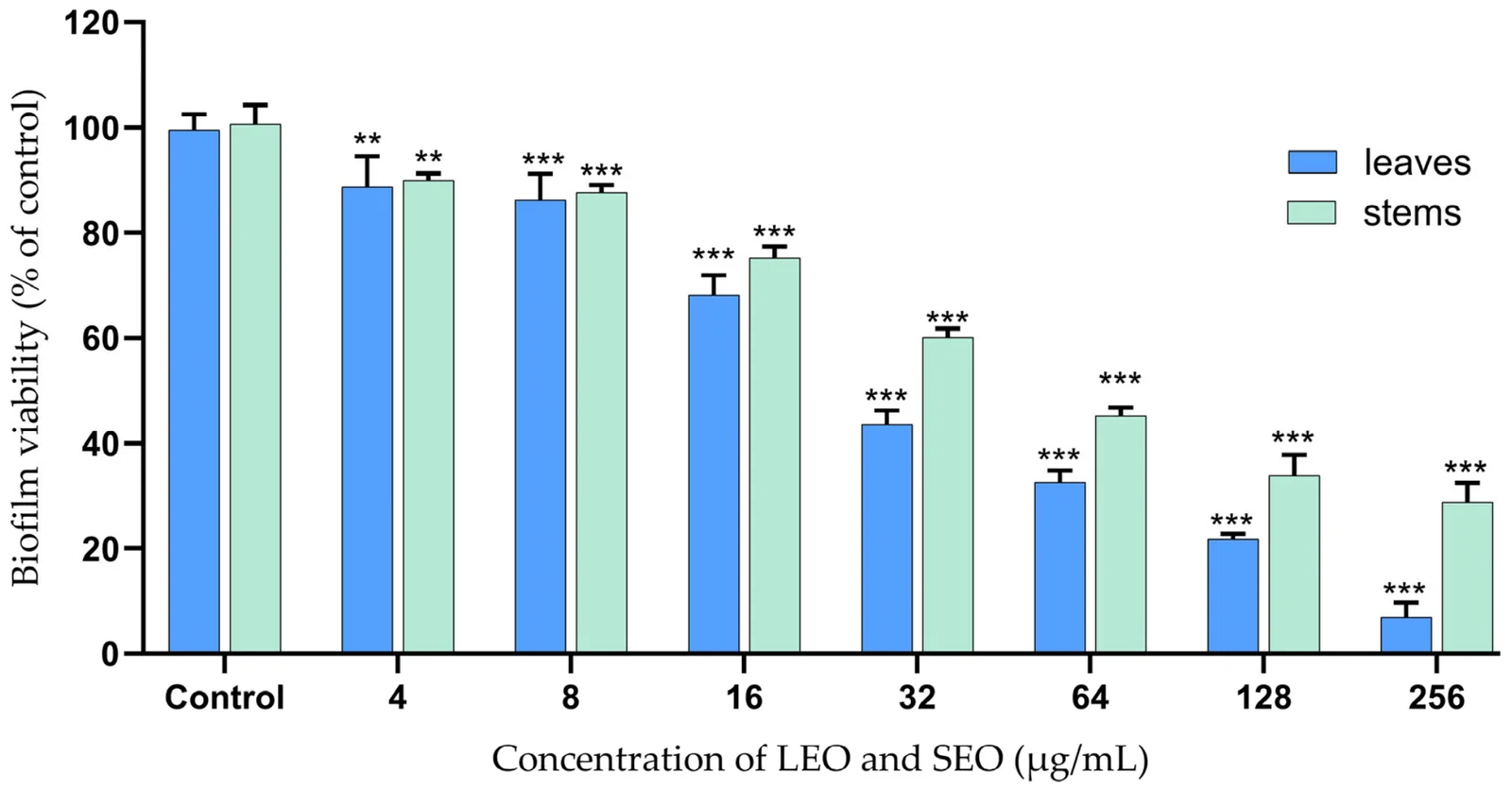
Percentage inhibition of *S. aureus* ATCC 25923 biofilm formation. Statistical significance of the differences (relative to the control) was established at *p* < 0.01 (**) and *p* < 0.001 (***).

The ability of *Dicranopteris pedata* leaf and stem essential oil to inhibit biofilm formation was evaluated using the crystal violet method [64], with chloramphenicol as the positive control. *S. aureus* (ATCC 25923) was selected as a model Gram-positive biofilm-forming pathogen because it is a leading cause of device-related, wound, and chronic biofilm-associated infections, readily forms biofilms under standard in vitro conditions, and is widely used as a model organism for antibiofilm testing of natural products [65]. Figure 2 revealed a dose-dependent response to LEO and SEO. Within the concentration range of 0–256 µg/mL, biofilm abundance (measured as OD_595_) possessed a gradual decreasing trend. Across all tested concentrations, LEO consistently exhibited significantly greater antibiofilm activity than SEO. The half-maximal inhibitory concentration (IC_50_) values (Table 5) demonstrated that LEO (31.53 ± 4.53 μg/mL) was approximately twice as potent as SEO (62.20 ± 9.36 μg/mL) in preventing biofilm formation. An IC_50_ of 1.12 ± 0.17 μg/mL was obtained for chloramphenicol. These results indicate that while both essential oils possess moderate anti-biofilm activity, LEO is significantly more effective than SEO. As biofilm characteristics are species- and strain-dependent, these results should not be extrapolated to other bacterial biofilms without further validation.

The biofilm formation inhibitory capacity of LEO over SEO may be partly explained by differences in their chemical composition, particularly the higher concentration of oxygenated monoterpenes in LEO, including its major constituent, linalool [56,66]. Oxygenated monoterpenes have been reported to act as effective quorum-sensing and biofilm inhibitors [56]. For instance, linalool has been reported to inhibit *S. aureus* growth and biofilm formation by over 80% at sub-MIC concentrations through the induction of reactive oxygen species (ROS) production and disruption of cell membrane integrity [67]. In addition, it has been reported that essential oils may exert their antibiofilm activity not only through bactericidal action but also by inhibiting quorum-sensing pathways that control biofilm formation [68]. The ability of linalool and other oxygenated monoterpenes to bind to quorum-sensing regulatory proteins and down-regulate expression of biofilm-associated genes has been documented, resulting in reduced bacterial adhesion and decreased extracellular polymeric substance production [69]. Accordingly, SEO’s lower content of these active oxygenated compounds may explain its weaker biofilm inhibition activity. Nevertheless, further studies are needed to confirm this hypothesis and to rule out contributions from other constituents, synergistic interactions, or growth inhibition.

Furthermore, LEO contained notable amounts of (*E*)-*β*-ionone (6.9%) and *α*-terpineol (3.9%). *β*-ionone has been shown to exhibit antimicrobial activity [53], and *α*-terpineol is a well-known biofilm inhibitor and antibiotic synergist [42,70,71,72,73]. Their presence probably contributes to the stronger anti-biofilm activity of LEO compared with SEO. The ability of monoterpenes to inhibit biofilm formation without necessarily killing bacterial cells is well documented; terpenes have been shown to interfere with the initial adhesion stage of biofilm development by down-regulating the expression of genes associated with initial cell adhesion and quorum sensing [56]. The observed anti-biofilm activity of *D. pedata* EOs, particularly LEO, deserves further investigation into their potential use as adjunctive therapies for biofilm-associated chronic infections, which are often refractory to antibiotic treatment.

### 2.5. MTT Assay for Cell Viability Inhibition

**Figure 3:**
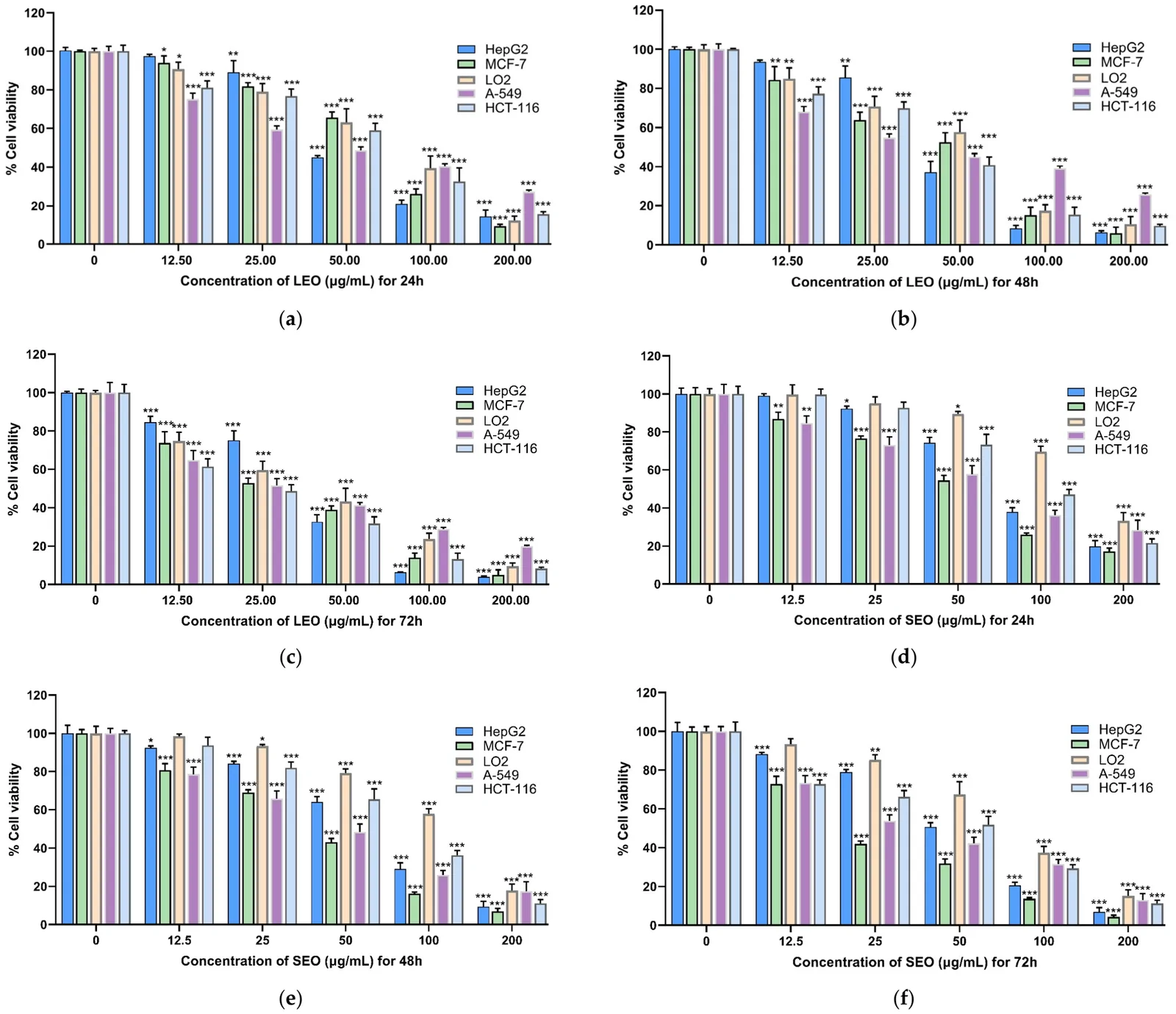
(**a**–**c**) Cell viability of LEO assessed at 24, 48, and 72 h, respectively. (**d**–**f**) Cell viability of SEO assessed at 24, 48, and 72 h, respectively. Statistical significance versus untreated control was defined as * *p* < 0.05, ** *p* < 0.01, and *** *p* < 0.001.

The cytotoxic effect of EOs was assessed against a panel of cultured human cancer cell lines (HepG2, MCF7, A-549, HCT-116) and normal LO2 cells via the MTT colorimetric assay. Figure 3 and Table 6 show that EOs possessed a marked dose- and time-dependent growth-inhibitory effect on the cell lines tested. As shown in Table 6, the IC_50_ values of LEO against HepG2, MCF7, A-549, HCT-116 and LO2 cells at 48 h were 43.63 ± 0.82, 40.14 ± 5.54, 39.17 ± 0.67, 36.86 ± 1.97 and 46.61 ± 3.52 μg/mL, whereas those for SEO were 62.32 ± 1.74, 38.03 ± 1.42, 43.94 ± 3.78, 67.34 ± 5.07 and 103.63 ± 2.64 μg/mL, respectively. The selectivity index (SI) was calculated as the ratio of the IC_50_ in the normal LO2 cell line to the IC_50_ in each cancer cell line and presented in Table 7. SEO exhibited greater in vitro selectivity than LEO, with higher SI values ranging from 1.54 to 2.72 at 48 h.

The cytotoxic screening results reveal that LEO exerted marked inhibitory effects on all cancer cells, with IC_50_ values ranging from 36.86 ± 1.97 μg/mL (HCT-116) to 43.63 ± 0.82 μg/mL (HepG2). Notably, LEO showed comparable activity against normal LO2 cells (46.61 ± 3.52 μg/mL), reflecting a lack of selective toxicity (SI ranging from 1.07 to 1.26). In comparison, SEO exhibited a more heterogeneous response profile: MCF7 was the most sensitive line (38.03 ± 1.42 μg/mL), followed by A-549 (43.94 ± 3.78 μg/mL), whereas HepG2, HCT-116, and LO2 were considerably less susceptible, with IC_50_ values of 62.32 ± 1.74, 67.34 ± 5.07, and 103.63 ± 2.64 μg/mL, respectively. Collectively, although LEO displayed superior potency across most tested cancer lines, SEO was notable for its selective action against cancerous versus normal cells (LO2; SI ranging from 1.54 to 2.72).

The cytotoxic effects could be partly associated with key compounds such as linalool and hexadecanoic acid (palmitic acid). Linalool has been reported to induce cell death in several cancer types [74]. Previous investigations have revealed that linalool exerts its anticancer effects through G0/G1 phase cell cycle arrest, accompanied by the induction of apoptosis in cancer cells [75]. Although linalool has been shown to possess low toxicity towards normal cells [76], the higher tolerance of LO2 cells to SEO, which contains a lower proportion of linalool than LEO, suggests that the favorable cytotoxicity of SEO cannot be attributed solely to its linalool content. Hexadecanoic acid could also contribute to the observed activity. It has demonstrated selective cytotoxicity against cancer cells while showing minimal toxicity towards normal fibroblasts, a property that aligns with the more selective profile of SEO [77,78,79]. However, essential oils are complex mixtures, and the observed activities may result from additive, synergistic, or antagonistic interactions among constituents [80].

## 3. Materials and Methods

### 3.1. Collection of the Botanical Material

The fresh plant *Dicranopteris pedata* was obtained in September 2023 from Lishui City in Zhejiang province of China. The voucher specimens (NO. 023011) were deposited at the Laboratory of Botany, Marine College, Shandong University, after being identified by Prof. Hong Zhao.

### 3.2. Dicranopteris Pedate EOs Preparation

The leaves and stems (800 g) of fresh *D. pedate* were subjected to hydrodistillation for 3 h using a Clevenger-type apparatus. The yields of leaf essential oil (LEO) and stem essential oil (SEO) were 0.11% and 0.07%, respectively, based on the weight of the dried plants. Anhydrous sodium sulphate was used to remove residual water from the essential oil. The dried oils were then stored in airtight dark vials and kept at 4 °C until they were analysed.

### 3.3. Analysis and Identification of EOs’ Constituents

An Agilent 7890B gas chromatograph, coupled with a Agilent 5977B mass-selective detector (electron ionization, 70 eV; Agilent Technologies, Santa Clara, CA, USA), was employed for the chemical characterization of the oil. Employing an HP-5MS fused-silica column (30 m × 0.25 mm i.d., 0.25 μm film; Agilent Technologies, Santa Clara, CA, USA) and helium (1.3 mL/min) as the carrier gas, the GC run was performed under the following thermal conditions: initial 60 °C (1 min), ramp to 230 °C at 5 °C/min, and hold for 5 min. The injector and detector temperatures were both maintained at 240 °C, while injections (0.2 μL of a 1% n-hexane solution) were made in split mode (1:50). Mass spectra were acquired over the *m*/*z* interval 50–550. GC analysis was performed on an Agilent 7890B gas chromatograph (Agilent Technologies, Santa Clara, CA, USA) equipped with a flame ionization detector (FID) and an HP-5MS fused-silica column (30 m × 0.25 mm i.d., 0.25 μm film thickness), under the same chromatographic conditions as those used for the GC/MS analysis.

Compound identification was achieved through mass spectral library searching against NIST 20, along with retention index determination (based on C_7_–C_30_ *n*-alkanes under parallel experimental conditions) and subsequent comparison with data from pure standards found in commercial mass spectra libraries and available in the literature [44,45,46].

### 3.4. Antibacterial Susceptibility Testing

Two Gram-negative and two Gram-positive bacterial strains were employed to assess the antibacterial efficacy of EO: *E. coli* ATCC 25922, *P. aeruginosa* ATCC 27853, *B. subtilis* ATCC 6633, and *S. aureus* ATCC 6538. These organisms were incubated on Mueller–Hinton (MH) agar (37 °C, 24 h). The bacterial suspensions were prepared in MH broth and adjusted to 0.5 McFarland standard (10^8^ CFU/mL), and a 1:100 dilution was then made to provide a final density of 10^6^ CFU/mL in the susceptibility test.

The determination of DP-EO’s Minimum Inhibitory Concentration (MIC) was carried out in 96-well microplates using the method of serial microdilutions, following the method described in the literature with slight modifications [47,48]. DP-EO was prepared in a solution of 5.12 mg/mL. The inoculum (100 μL) was added to each well, with the first well containing 100 μL of DP-EO solution, and twofold dilutions of DP-EO were made in 96-well microtiter plates with concentrations ranging from 5.12 to 0.01 mg/mL. In each microdilution plate, the combination of medium and strain served as the positive growth control, whereas medium only functioned as the negative growth control. The 96-well plates containing bacteria were incubated at 36 °C for 24 h, after which 20 µL of 1% TTC (2,3,5-triphenyltetrazolium chloride) was added to each well to indicate bacterial growth. The MIC corresponded to the lowest concentration that yielded no colour change (i.e., no growth). The MIC was defined as the lowest concentration at which bacterial growth was inhibited. For MBC measurement, 100 μL of culture medium from wells (without any microbial growth) was subcultured onto MHA plates and further incubated for 24 h. The MBC was defined as the lowest concentration of essential oils at which there was no visible growth on agar plates after subculture.

### 3.5. Synergistic Interaction

The synergistic antibacterial effects between DP-EO and selected antibiotics (chloramphenicol and streptomycin) were determined by the checkerboard dilution method [57]. Based on the MIC values of DP-EO and antibiotics obtained from the previous experiments, each antibiotic and DP-EO were diluted in Müller–Hinton Broth (MHB) to serial two-fold dilutions (from 4-fold to 1/64 of their MICs), and 50 μL of each dilution was placed in the 96-well microplates. The checkerboard is composed of columns, with each column containing an equal amount of DP-EO being diluted along the x-axis. Similarly, the rows contain an equal amount of the antibiotic being diluted along the y-axis. Therefore, each well in the checkerboard represents a distinct concentration of the tested combination. Each well was filled with 100 μL of bacterial suspension, and the microtiter plate was then incubated at 37 °C for 24 h. After this, 20 μL of TTC (1% *w*/*v*) was added to the wells and further incubated at 36 °C for 30 min. The wells located at the interface where growth meets no growth were identified. FICI was then determined using the following equation to assess the effect of the interaction:

$$\mathrm{FICI}\;=\;\frac{\mathrm{Combined}\;\mathrm{MIC}\;\mathrm{of}\;\mathrm{DP}\text{-}\mathrm{EO}\;}{\mathrm{Individual}\;\mathrm{MIC}\;\mathrm{of}\;\mathrm{DP}\text{-}\mathrm{EO}}+\;\frac{\mathrm{Combined}\;\mathrm{MIC}\;\mathrm{of}\;\mathrm{antibiotic}}{\mathrm{Individual}\;\mathrm{MIC}\;\mathrm{of}\;\mathrm{antibiotic}}$$

The findings were elucidated as follows: synergistic, FICI ≤ 0.5; additive, 0.5 < FICI ≤ 0.75; indifferent, 0.75 < FICI ≤ 2; no effect, FICI ≥ 2 [58].

### 3.6. Antibiofilm Activity

The inhibition of biofilm formation by DP-EO was evaluated using the 96-well microtiter plate-based crystal violet staining method [81]. In this assay, 100 μL of *S. aureus* (ATCC 25923) suspension (adjusted to a final concentration of approximately 1 × 10^6^ CFU/mL per well) and 100 μL of two-fold serial dilutions of the EO were added into each well. The untreated bacterial suspension (without EO) served as the growth control for biofilm formation, while wells containing only culture medium with the corresponding solvent were used as blank controls to subtract background absorbance. The plates were sealed with sterile breathable films and incubated at 37 °C for 24 h. After that, the culture medium was carefully removed, and the wells were washed gently with phosphate-buffered saline three times to avoid disrupting the adherent biofilm. The established biofilms were fixed with 200 μL of methanol per well for 15 min. Following the removal of methanol and air-drying, adherent biofilms were stained with 200 μL of 1% (*w*/*v*) crystal violet solution for 20 min, and then rinsed with distilled water. The crystal violet retained by the biofilms was dissolved in 200 μL of 95% ethanol. Finally, the absorbance was measured at 570 nm using an ELISA microtiter plate reader (Epoch; BioTek Instruments, Inc., Winooski, VT, USA).

### 3.7. Cytotoxic Activity

A panel of human cancer cell lines—HepG2 ATCC HB-8065 (liver), MCF-7 ATCC HTB-22 (breast), A-549 ATCC CCL-185 (lung), and HCT-116 ATCC CCL-247 (colon)—along with the normal hepatic line LO2 (RRID CVCL_6926) was acquired from the Shanghai Institute for Biological Sciences (SIBS), located in Shanghai, China. RPMI 1640 medium, supplemented with 2 mM glutamine, 10% FCS, and 100 U/mL each of penicillin and streptomycin, was used to grow the cells, which were then maintained at 37 °C in a 5% CO_2_ incubator.

The cytotoxicity of DP-EO was measured by using a modified MTT assay [82]. Each well received 5 × 10^4^ cells in 100 µL of culture medium, which were then incubated for 24 h to allow attachment. The DP-EO was first dissolved in DMSO and diluted in culture medium; a series of two-fold dilutions of DP-EO or positive control (doxorubicin) were then added to triplicate wells at a concentration range of 200–12.5 μg/mL and were incubated for 24, 48 and 72 h, respectively. The final concentration of DMSO in the test medium and controls was 1% (*v*/*v*). Cell viability was measured using the MTT colorimetric assay. Absorbance was measured at 570 nm, and cell viability was calculated relative to the untreated control to determine the cytotoxic effects of DP-EO.

### 3.8. Statistical Analysis

Triplicate determinations were performed for each experiment, with the results given as mean ± SD. For statistical assessment, IBM SPSS Statistics (ver. 31) was employed, and a one-way ANOVA followed by Dunnett’s post hoc test was applied to identify significant differences from the control (defined as *p* < 0.05). All graphical illustrations were produced with GraphPad Prism 10.

## 4. Conclusions

Herein, we report the first systematic analysis of the phytochemical constituents and in vitro biological activities of EOs derived from the *Dicranopteris pedate* leaves (LEO) and stems (SEO). GC-MS examination indicated that LEO has a high content of oxygenated monoterpenes (38.9%), with linalool (21.0%) as the dominant constituent, while SEO contains lower levels of these bioactive compounds (18.9% oxygenated monoterpenes; 4.5% linalool) and higher proportions of fatty acid derivatives. Consistent with their chemical profiles, LEO exhibited superior antibacterial effect with MIC values two- to four-fold lower than those of SEO against tested bacterial strains. Notably, LEO demonstrated exceptionally potent synergy with chloramphenicol and streptomycin against *P. aeruginosa*, reducing antibiotic MICs by up to 1000-fold. LEO also showed stronger anti-biofilm activity against *S. aureus* (IC_50_ = 31.53 ± 4.53 μg/mL) compared with SEO (62.20 ± 9.36 μg/mL). Regarding cytotoxicity, LEO was markedly active against all tested cancer cell lines, whereas SEO displayed a more selective profile, being less toxic to normal LO2 cells. Taken together, *D. pedata* leaf essential oil—particularly owing to its high oxygenated monoterpene content—represents a promising natural source of antibacterial, synergistic, and anti-biofilm agents, with stem oil offering potential advantages in selective anticancer applications. However, as the evidence is limited to chemical characterization and in vitro bioactivity assays, further mechanistic studies, evaluation against clinically relevant and resistant strains, and in vivo investigations are necessary before the application in pharmaceutical practice.

## Figures and Tables

**Table 2:** MIC and MBC values of the *D. pedate* leaves and stems EOs.

Microorganism	MIC (μg/mL)			MBC (μg/mL)		
	LEO	SEO	Chl	LEO	SEO	Chl
*B. subtilis* ATCC 6633	20.00	80.00	2.00	20.00	160.00	4.00
*S. aureus* ATCC 6538	160.00	320.00	4.00	320.00	640.00	8.00
*E. coli* ATCC 25922	640.00	640.00	4.00	640.00	640.00	32.00
*P. aeruginosa* ATCC 27853	320.00	640.00	32.00	1280.00	1280.00	256.00

Chl: Chloramphenicol (positive control).

**Table 3:** Results of synergism test of *D. pedate* EO/chloramphenicol combinations.

Strain	Combination	Individual MIC (μg/mL)	Combined MIC (μg/mL)	FICI
*B. subtilis* ATCC 6633	LEO	20.00	5.00	0.28 (S)
	Chl	2.00	0.06	
	SEO	80.00	20.00	0.38 (S)
	Chl	2.00	0.25	
*S. aureus* ATCC 6538	LEO	160.00	20.00	0.25 (S)
	Chl	4.00	0.50	
	SEO	320.00	80.00	0.31 (S)
	Chl	4.00	0.25	
*E. coli* ATCC 25922	LEO	640.00	160.00	0.50 (S)
	Chl	4.00	1.00	
	SEO	640.00	160.00	0.50 (S)
	Chl	4.00	1.00	
*P. aeruginosa* ATCC 27853	LEO	320.00	20.00	0.06 (S)
	Chl	32.00	0.03	
	SEO	640.00	80.00	0.25 (S)
	Chl	32.00	4.00	

Chl: chloramphenicol. S, synergy.

**Table 4:** Results of synergism test of *D. pedate* EO/streptomycin combinations.

Strain	Combination	Individual MIC (μg/mL)	Combined MIC (μg/mL)	FICI
*B. subtilis* ATCC 6633	LEO	20.00	5.00	0.31 (S)
	Str	1.00	0.06	
	SEO	80.00	10.00	0.38 (S)
	Str	1.00	0.25	
*S. aureus* ATCC 6538	LEO	160.00	5.00	0.28 (S)
	Str	2.00	0.50	
	SEO	320.00	40.00	0.38 (S)
	Str	2.00	0.50	
*E. coli* ATCC 25922	LEO	640.00	80.00	0.63 (A)
	Str	0.50	0.25	
	SEO	640.00	160.00	0.51 (A)
	Str	0.50	0.13	
*P. aeruginosa* ATCC 27853	LEO	320.00	10.00	0.05 (S)
	Str	2.00	0.03	
	SEO	640.00	20.00	0.16 (S)
	Str	2.00	0.25	

Str, streptomycin. S, synergy. A, additivity.

**Table 5:** Biofilm formation inhibition effect of EOs from *D. pedata* leaves and stems against *Staphylococcus aureus* ATCC 25923.

Strains	LEO IC_50_ (μg/mL)	SEO IC_50_ (μg/mL)	Chl IC_50_ (μg/mL)
*Staphylococcus aureus* ATCC 25923	31.53 ± 4.53	62.20 ± 9.36	1.12 ± 0.17

**Table 6:** Cytotoxicity (IC_50_ μg/mL) of LEO and SEO.

	Sample	24 h	48 h	72 h
HepG2	LEO	56.70 ± 2.89	43.63 ± 0.82	35.33 ± 3.10
	SEO	86.14 ± 2.40	62.32 ± 1.74	48.24 ± 0.48
	Doxorubicin	1.46 ± 0.08	1.09 ± 0.08	0.433 ± 0.07
MCF-7	LEO	61.26 ± 1.37	40.14 ± 5.54	28.68 ± 2.66
	SEO	54.71 ± 2.36	38.03 ± 1.42	23.90 ± 1.78
	Doxorubicin	1.56 ± 0.03	0.79 ± 0.02	0.39 ± 0.06
LO2	LEO	66.48 ± 8.87	46.61 ± 3.52	35.07 ± 5.10
	SEO	147.26 ± 9.94	103.63 ± 2.64	73.35 ± 6.90
	Doxorubicin	1.80 ± 0.29	0.46 ± 0.02	0.55 ± 0.12
A-549	LEO	50.63 ± 3.81	39.17 ± 0.67	28.86 ± 1.77
	SEO	67.61 ± 5.51	43.94 ± 3.78	36.81 ± 5.04
	Doxorubicin	1.04 ± 0.07	0.85 ± 0.05	0.33 ± 0.01
HCT-116	LEO	57.26 ± 1.29	36.86 ± 1.97	21.52 ± 2.09
	SEO	94.33 ± 4.55	67.34 ± 5.07	41.98 ± 4.14
	Doxorubicin	1.33 ± 0.15	0.57 ± 0.02	0.48 ± 0.06

Doxorubicin served as a positive control. Data are presented as means ± SD from three independent replicates.

**Table 7:** Selectivity index (SI) of LEO and SEO against different cell lines.

Sample	Time	Cell Lines			
		HepG2	MCF-7	A-549	HCT-116
LEO	24 h	1.17	1.09	1.31	1.16
	48 h	1.07	1.16	1.19	1.26
	72 h	0.99	1.22	1.22	1.63
SEO	24 h	1.71	2.69	2.18	1.56
	48 h	1.66	2.72	2.36	1.54
	72 h	1.52	3.07	1.99	1.75

SI = IC_50_ (normal cells)/IC_50_ (cancer cells).

## Data Availability

The data are contained in this article.
